# An In-Depth Investigation into the Physicochemical, Thermal, Microstructural, and Rheological Properties of Petroleum and Natural Asphalts

**DOI:** 10.3390/ma9100859

**Published:** 2016-10-21

**Authors:** Nader Nciri, Jeonghyun Kim, Namho Kim, Namjun Cho

**Affiliations:** 1 Department of Energy, Materials, and Chemical Engineering, Korea University of Technology and Education, 1600 Chungjeol-ro, Byeongcheon-myeon, Dongnam-gu, Cheonan-City, Chungnam-Province 330-708, Korea; nader.nciri@koreatech.ac.kr; 2 Department of Architectural Engineering, Korea University of Technology and Education, 1600 Chungjeol-ro, Byeongcheon-myeon, Dongnam-gu, Cheonan-City, Chungnam-Province 330-708, Korea; jhk16@koreatech.ac.kr (J.K.); nhkim@koreatech.ac.kr (N.K.)

**Keywords:** petroleum asphalt, natural asphalt, chemical compositions, microstructures, thermo-analytical behaviors, physico-rheological properties

## Abstract

Over the last decade, unexpected and sudden pavement failures have occurred in several provinces in South Korea. Some of these failures remain unexplained, further illustrating the gaps in our knowledge about binder chemistry. To prevent premature pavement distress and enhance road performance, it is imperative to provide an adequate characterization of asphalt. For this purpose, the current research aims at inspecting the chemistry, microstructure, thermal, and physico-rheological properties of two types of asphalt, namely petroleum asphalt (PA) and natural asphalt (NA). The binders were extensively investigated by using elemental analysis, thin-layer chromatography with flame ionization detection (TLC-FID), matrix-assisted laser desorption ionization time-of-fight mass spectroscopy (MALDI-TOF-MS), Fourier transform infrared spectroscopy (FT-IR), Raman spectroscopy (RS), Nuclear magnetic resonance spectroscopy (^1^H-NMR), ultraviolet and visible spectroscopy (UV-VIS), X-ray diffraction (XRD), scanning electron microscopy (SEM), thermogravimetric analysis (TGA), differential scanning calorimetry (DSC), penetration, softening point, ductility, and viscosity tests. The findings of this research have revealed the distinct variations between the chemical compositions, microstructures, and thermo-rheological properties of the two asphalts and provided valuable knowledge into the characteristics of the binders. Such insight has been effective in predicting the performance or distress of road pavement. This paper will, therefore, be of immediate interest to materials engineers in state highway agencies and asphalt industries.

## 1. Introduction

For enhancing the sturdiness and performance of roads, the Korean Pavement Research Program (KPRP) was founded. The motivation behind this program was to provide safer means of passage to both motorists and highway workers. In order to acquire higher pavement performance, the KPRP utilizes its funds in developing the desired performance-based asphalt binder and mixture specifications through laboratory methods. For attaining beneficial characterization and specifications of asphalt, it is imperative to first comprehend its fundamental properties. At present, some critical gaps have been observed in the specifications of the asphalt binders; they are not adequately recognized as regards their chemical contribution to the pavement performance. The chemical compositions of the asphalts are found to control its physical characteristics in the final analysis [1,2], and yet most scientists deny the need for chemical compositions in determining asphalt specifications.

It is a known fact that asphalts can be equipped with similar physical characteristics while differing in terms of SARA (saturates, aromatics, resins, and asphaltenes) combinations [3]. While constructing a highway, the materials used are dependent on their physical and rheological properties. Such properties comprise of penetration, ductility, softening point, viscosity, etc. [4]. Nevertheless, roads are subjected to rutting, undesirable potholes, and cracking due to the deployment of unsatisfactory characterized binders. The inability to comprehend the binder chemistry and its relation with the pavement performance is evident in the escalating amounts of unexpected pavement failures across various provinces in the past few years. Also, some of these misgivings are left unexplained. Therefore, examining and detecting the properties of the asphalt binder that result in high pavement performance will benefit users, as the information can be exploited in determining the binders as per the performance criteria. When applying this theory in practice, it is evident that the asphalt will have to be modified for generating binders with consistent and reliable performance characteristics.

This research aims at revealing all the aspects of asphalt chemistry that will enable highway agencies to develop high-quality pavement through new specifications for asphalt cement. For this purpose, the current study will recognize the characteristics of the available materials along with the distinct parameters in their chemical compositions that will assist in the manufacturing of the desired asphalt cement by controlling their chemical composition. One efficient method to enhance the asphalt is blending the available asphalt sources with additives on the basis of the knowledge acquired from their chemical compositions. Another method is to modify the asphalt through the implementation of designing processes, where the desired properties will be instilled in the asphalt. The knowledge about the asphalt composition will be helpful in identifying the necessary components for modifying the asphalt and producing an asphalt aggregate that will be superior in terms of durability and performance in a specific environment. It will also be useful in detecting failures and constructing solutions to rectify damage. Therefore, this research is anticipated to assist asphalt technologists and highway engineers.

This investigation utilizes petroleum asphalt (PA) obtained from an anonymous refining company in South Korea to examine the characteristics and specifications of the asphalt with the help of various analytical techniques. These analytical methods are elemental analysis (EA), thin-layer chromatography with flame ionization detection (TLC-FID), Raman spectroscopy, FT-IR spectroscopy, Nuclear magnetic resonance spectroscopy (^1^H-NMR), UV-Vis spectroscopy, scanning electron microscopy (SEM), matrix-assisted laser desorption ionization time-of-fight mass spectroscopy (MALDI-TOF-MS), X-ray diffraction (XRD), differential scanning calorimetry (DSC) analysis (for analyzing the glass transition temperature (T_g_)), and thermogravimetric analysis (TGA) (for estimating boiling distributions). A battery of standard rheological techniques was also applied such as penetration, viscosity, softening point, and ductility. In addition, structural comparisons with natural asphalt (NA) are made for assessing the findings within the framework of enhanced materials.

## 2. Materials and Methods

### 2.1. Materials

For the purposes of the experiments, petroleum asphalt (PA) and natural asphalt (NA) were acquired from an anonymous refining company in South Korea, and the asphalt quarry near Vernal, UT, USA, respectively.

PA is manufactured by refining a crude oil through atmospheric distillation followed by vacuum distillation. The atmospheric distillation is completed at temperatures in the range of 300–320 °C. The atmospheric residue is then sent to a vacuum distillation unit that operates around 320 °C. Afterwards, the bottom product (PA) was subjected to various types of chemical and physical treatments.

The NA consists of a mixture of bitumen and minerals of the following composition: soluble bitumen (56 wt %), clay minerals (8 wt %), and moisture (0 wt %). It is a sticky, black, and semisolid liquid at room temperature. NA is extracted after heating in a furnace at 235 °C. 

The following methods and analysis were performed for estimating the association between the asphalt rheological properties, composition, and microstructure. All the solvents and reagents used in the assay procedures were analytical grade.

### 2.2. Analytical Methods

#### 2.2.1. Elemental Analysis (EA)

For determining the quantity of C, H, N, O, and S in the asphaltic materials, a Thermo Fisher Scientific™ Flash 2000 organic elemental analyzer (Thermo Fisher Scientific Inc., Darmstadt, Germany) was utilized to perform the elemental analysis. 

#### 2.2.2. Thin-Layer Chromatography with Flame Ionization Detection Procedure (TLC-FID)

Iatroscan MK-6 analyzer (Iatron Laboratories Inc., Chiyoda, Tokyo, Japan) was utilized to perform TLC-FID following the procedure defined in a published paper [5]. For the experiment, 80 mg of asphalt was added to 4 mL toluene to prepare a sample with a concentration of 2% (w/v). The chromarods were then washed and stimulated in the FID-flame. Then, 1 µL of sample solution was speckled onto the chromarods with the help of a spotter. To segregate the asphalt into the SARA (i.e., saturates, aromatics, resins, and asphaltenes) compositions, a three-stage process was deployed. In the first stage, the *n*-heptane experienced an expansion in chromarods to 100 mm. In the second stage, the toluene/*n*-heptane (80/20 by volume) was expanded to 50 mm, and in the last stage the dichloromethane/methanol (95/5 by volume) was expanded to 25 mm. Once these developments were made, the solvents were vaporized in an oven at 80 °C. After that the chromarods were scanned in a TLC-FID analyzer (Iatron Laboratories Inc., Chiyoda, Tokyo, Japan).

#### 2.2.3. Matrix-Assisted Laser Desorption Ionization Time-of-Flight Mass Spectroscopy (MALDI-TOF-MS)

MALDI-TOF-MS test was applied to determine the mean molar mass values with the assistance of a Bruker Autoflex III (Bruker Daltonik GmbH, Bremen, Germany) with an N_2_ (50 Hz) laser, in reflectron mode. The target plate was positively charged at 20 kV. The mass range was scanned from 0 to 2500 *m/z*. One milliliter of THF (tetrahydrofuran) was taken and the asphaltic samples were dissolved into it. They were then speckled onto the target plate devoid of the matrix. The samples were dried once the spotting was done and before the analysis was instigated. Dithranol (DT; 1, 8-dihydroxy-9, 10-dihydroanthracen-9-one) was used as the matrix. A delayed extraction mode, an extraction delay time of 500 ns, a manual acquisition control, and a grid voltage of 70% were set as the operating parameters. 

#### 2.2.4. Fourier Transform Infrared Spectroscopy (FT-IR)

KBr pellets were utilized to record the Bruker equinox 55 spectrometer (Bruker Daltonik GmbH, Bremen, Germany) in the area of 4000–650 cm^−1^ for determining the FT-IR spectra of different fractions of asphalt.

#### 2.2.5. Raman Spectroscopy (RS)

A Nanofinder FLEX G Raman microscope (Tokyo Instruments Inc., Edogawa, Tokyo, Japan) was utilized to conduct Raman spectroscopy by examining the results at high resolution. Prolonged scans from 200 to 3600 cm^−1^ were executed using a typical exposure time and an accumulation of 3 s/20 time. The microscope was helpful in concentrating on the excitation laser beam (green line of an argon laser *λ* = 532 nm) onto the material. Twenty measurements were taken for each sample.

#### 2.2.6. Nuclear Magnetic Resonance Spectroscopy (^1^H-NMR)

Qualitative proton ^1^H-NMR spectroscopy analysis was conducted by deploying a Varian XL-300 NMR spectrometer (Agilent Technologies Inc., Santa Clara, CA, USA). This instrument was set at a frequency of 300.13 MHz to perform the test for proton. The asphaltic samples were then dissolved in Deuteriochloroform (CDCl_3_). The data for the proton was obtained by setting the acquisition time at 4.56 s and sweep width at 3591 Hz with no recycle delay. The proton spectra were referenced to the deuterated chloroform (CDCl_3_) resonance at 7.28 ppm. 

#### 2.2.7. Ultraviolet and Visible Spectroscopy (UV-VIS)

Spectra were recorded on a JASCO V-730 spectrophotometer (JASCO International Co., Ltd., Hachioji, Tokyo, Japan) in the wavelength range 240‒600 nm, with a bandwidth of 2 nm, a scanning speed of 100 nm/min, and a response of 2 s. Measurements were conducted in a 1-cm quartz cuvette at ambient temperature and pressure. Samples for optical studies were prepared by dilution of the asphalt with a chemically pure grade dichloromethane (CH_2_Cl_2_) to a final concentration of 0.001%. 

#### 2.2.8. X-ray Diffraction (XRD)

For this experiment, a high-power X-ray diffractometer (D8 Advance, Bruker AXS GmbH, Berlin, Germany) was used at 12 kW. This instrument comprised of a rotating copper anode, a scintillation counter, and a graphite monochromator. The asphaltic samples were subjected to this analysis, where the diffraction patterns (5° ≤ 2θ ≤ 70°) were recorded at room temperature using CuKα radiation (λ = 1.54055 Å) at a scanning rate of 1 deg·min^−1^ and a step size of 0.02 (2θ).

#### 2.2.9. Scanning Electron Microscopy (SEM)

A CX-100 scanning electron microscope (JEOL Ltd., Musashino, Akishima, Japan) was employed in acquiring the morphology of the asphalt. These samples were layered with gold for 100 s in vacuum with the help of a JFC-1600 fine coater (JEOL Ltd., Musashino, Akishima, Japan).

#### 2.2.10. Thermogravimetric Analysis (TGA)

TA Instruments, i.e., TGA Q500 V20.13 Build 39 thermobalance (TA Instruments, Hüllhorst, Germany) was used to conduct the thermogravimetric analysis. The test was performed under air atmosphere. Those samples with 10 to 20 mg of mass were heated at 10 °C/min from room temperature to 700 °C. 

#### 2.2.11. Differential Scanning Calorimetry (DSC)

Perkin-Elmer 7500 DSC (PerkinElmer Inc., Rodgau, Germany) was utilized to perform the DSC tests. The instrument was adjusted at a temperature of 20 °C/min against indium. Argon and helium were utilized as the purging gases when the tests started at −60 °C and −100 °C. At 30 °C, the samples were kept in the sample cell and consequently cooled down to −60 °C or −100 °C at a cooling rate of 200 °C/min. For approximately 15 min the samples were kept at the low temperature to obtain adequate results. Afterwards, they were heated to 100 °C at a heating rate of 20 °C/min. At this temperature, the readings observed through the DSC thermogram were rendered as the first scan. After this observation, the sample was cooled at a rate of 200 °C/min from 100 °C to its starting temperature (−60 °C or −100 °C) and again held for about 15 min before being reheated to 100 °C at a heating rate of 20 °C/min. The recording during this second heating scan was rendered as the second scan. Perkin-Elmer’s software was then used to convert the DSC thermogram data into ASCII format to conduct a comprehensive analysis on an IBM PS/2 computer. This research deployed a new computer program to estimate and determine the calorimetric parameters.

### 2.3. Determination of Rheological Properties

Penetration, viscosity, softening point, and ductility were the four tests that revealed the physical or rheological properties of the recovered asphalt. To conduct the penetration test, a temperature of 25 °C, loading weight of 100 g and needle penetrating time of 5 s were taken. These parameters were consistent with ASTM D5-IP49, whereas the softening point test was conducted as per the ASTM D36; ASTM D4402 was referred to for the viscosity test. A Brookfield Model DV-I viscometer (Brookfield AMETEK, Middleborough, MA, USA) was utilized to perform the test for viscosity with a thermocel temperature control system. The spindle deployed for determining the viscosity behavior of asphalt in different conditions was SC-27. It was kept at a temperature of 135 °C (field condition) and 180 °C (plant mixing condition). When considering the ductility test of the asphalt, ASTM D113 was used. The ductility property is defined as the distance to which the asphalt will elongate before breaking when two ends of the material are stretched at a particular speed and temperature. The test was performed at a temperature 25 °C with 5 cm/min speed.

## 3. Results and Discussion

As mentioned before, the current research aims at providing valuable insights into the chemical compositions of the asphalts and their impact on its physical properties. This information will be useful in generating adequate and efficient asphalt binders for road pavements. Also, this research provides new basic tools to study asphalt chemistry. However, to assess the importance of the findings obtained in a particular asphalt binder product are outside the scope of this work. 

### 3.1. Elemental Analysis (EA)

Table 1 demonstrates the distinct chemical compositions of both the asphalts (PA and NA) observed from the elemental analysis. From the analysis, it was found that asphalt constitutes higher amounts of hydrocarbons (79.01~87.66 wt % carbon and 9.11~10.14 wt % hydrogen) and lower amounts of heteroatoms (traces of up to 1 wt % nitrogen, traces of up to 6 wt % sulfur, and traces of up to 1.40 wt % oxygen). There is no difference between the H/C values of PA (1.38) and NA (1.38). This low atomic H/C ratio establishes their aromatic character.

### 3.2. Thin-Layer Chromatography with Flame Ionization Detection (TLC-FID)

**Figure 1:**
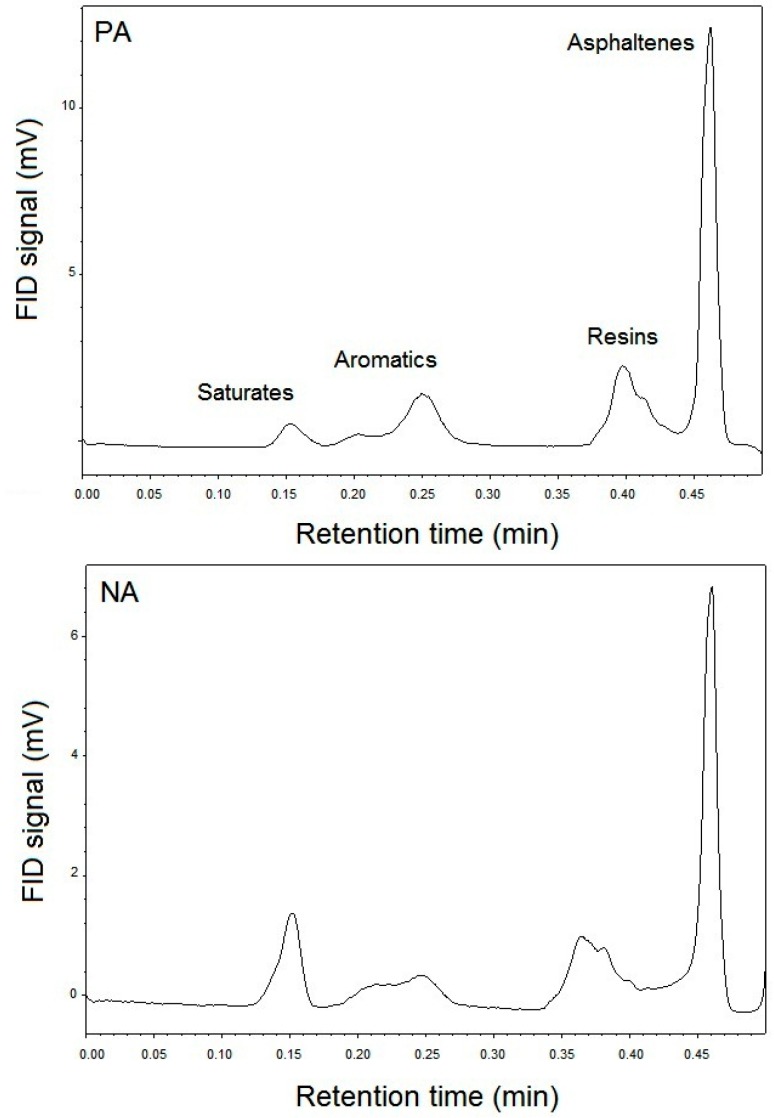
Iatroscan chromatographs of PA and NA.

It should be noted that the asphalts have an exceptionally complex chemical composition. Solvent precipitation and chromatography are two of the techniques that have been efficient for fractionation of asphalts. For the current research, the TLC-FID technique is utilized. Figure 1 demonstrates the classic TLC-FID chromatograms of asphalts. The four peaks visible in Figure 1 depicts the SARA compositions (saturates, aromatics, resins, and asphaltenes). Table 2 demonstrates the generic fractions of the asphalt material by normalizing the area. With the help of the standard composition, the colloidal instability index (I_C_) is also estimated and presented in the table.

The difference between the chemical compositions of PA and NA is evident in Table 2. It is observed that PA and NA have different values of SARA fractions, which results in varied colloidal instability index (I_C_) values. When considering the group composition, the contents of aromatics hydrocarbon (18.97 wt %) are observed to be higher in PA. On the other hand, the contents of saturates (4.40 wt %) in PA are observed to be lower than NA. 

When considering the stability aspect, it is observed that PA is slightly more stable than NA as it has a low I_C_ value. As seen in Table 2, the values of saturates and asphaltenes are low in PA, which is why it has acquired higher stability. Also, the reason behind its stability is that PA has superior peptizability due to the smaller aggregation of asphaltenes’ micelle.

Otherwise, the saturates and asphaltenes are the lowest and highest polarity components of an asphalt, respectively, and the solubility of the asphaltenes in a colloidal system is enhanced by the presence of intermediate polarity species such as aromatics and resins. Thus, the higher the I_C_, the less stable the overall system is.

### 3.3. Matrix-Assisted Laser Desorption Ionization Time-of-Flight Mass Spectroscopy (MALDI-TOF-MS)

**Figure 2:**
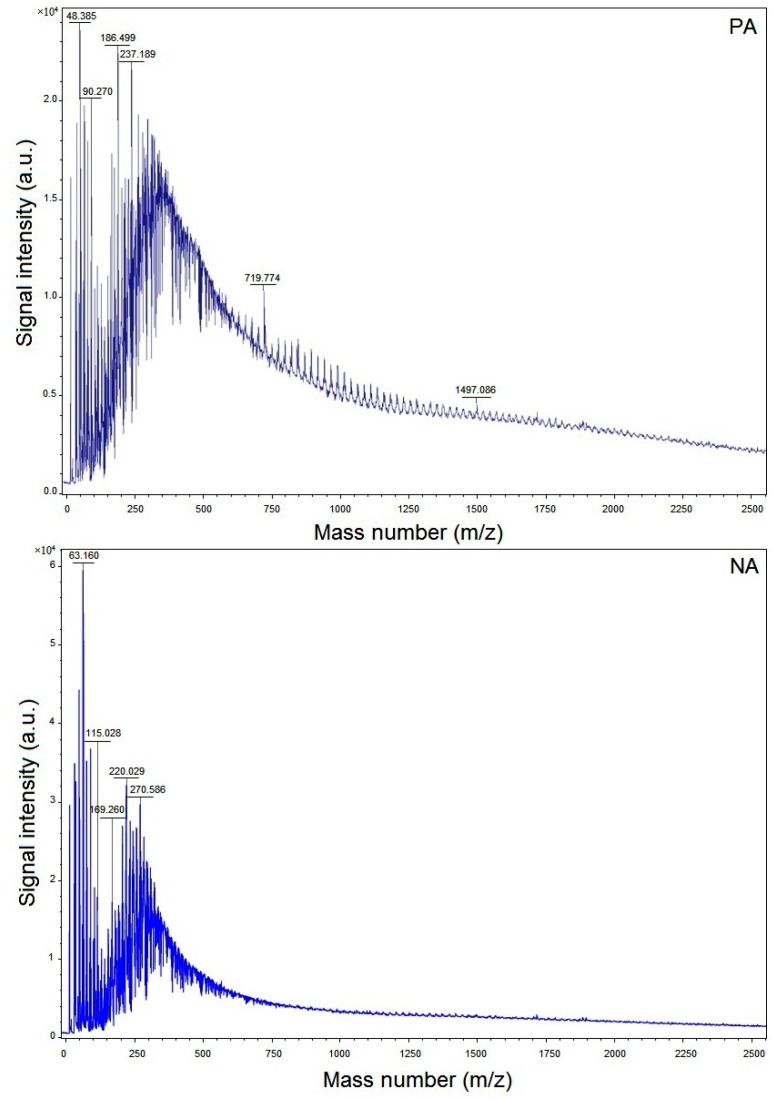
MALDI-TOF-MS spectra of PA and NA and respective average molar masses.

Molar mass is one attribute of a molecule that results in its solubility. With the increase in molar mass of molecules with a similar structure, their solubility decreases. When the asphalt fractions are separated, it is observed that those that are highly insoluble have higher molar mass. The classic mass spectra for both PA and NA are illustrated in Figure 2. Due to the oligomeric nature of the asphalts, they are segregated into four regions: monomers from *m*/*z* 200 to 400, dimers from *m*/*z* 400 to 650, trimers from *m*/*z* 650 to 950, and tetramers from *m*/*z* 950 to 1600. The samples present significant signal intensity up to 48.38 ×10^4^~63.16 ×10^4^ a.u. It is revealed from the MALDI-TOF-MS analysis that the PA is comprised of molecules with higher molar mass (328 Da) than NA, which had 252 Da. These results are obtained from the comparison of PA and NA bitumens and are presented in Figure 2. The reason behind these results is attributed to the higher heteroatom content and lower H/C ration in PA. On the other hand, when compared with the asphalt fractions, PA has high aromatics and low saturates.

### 3.4. FT-IR Analysis

**Figure 3:**
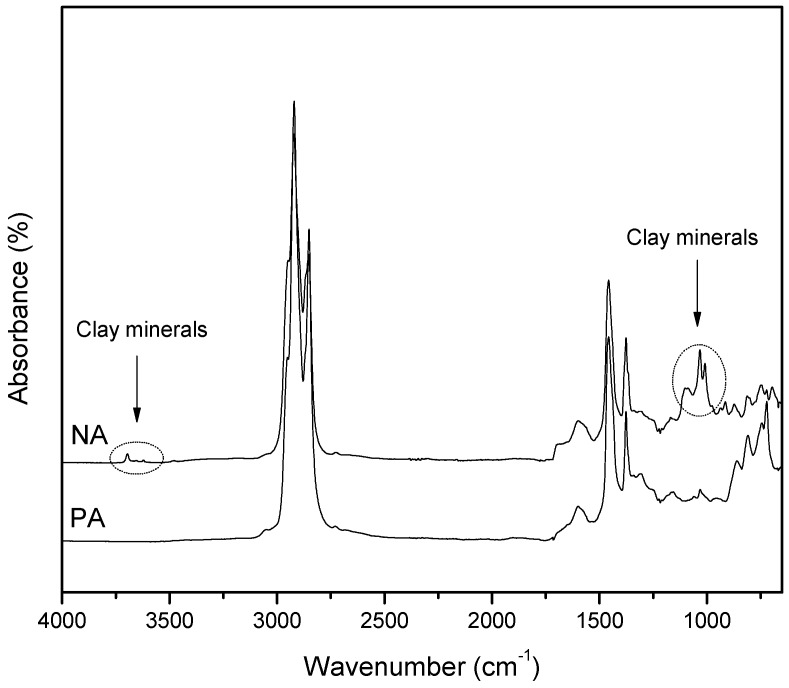
FT-IR spectra of NA and PA.

In analytical chemistry, FT-IR is found to be one of the most flexible and resourceful techniques. It is efficient at providing valuable information about the chemical functional groups of asphalts in complex solids [6]. Figure 3 demonstrates the infrared spectra of PA and NA. For the scenario of NA, the characteristics of IR spectra of clay minerals are clearly visible in Figure 3, where Si‒OH and absorbed water on clay are illustrated through the band near 3650 cm^−1^. In Figure 3, the broad band around 1600 cm^−1^ is assigned to the ring vibration of aromatic compounds. Whereas the band at 1083 cm^−1^ represents the anti-symmetric unfolded vibration of Si‒O‒Si [7], the other bands are detected within the range of 1000 and 650 cm^−1^, which represent the characteristics of clay materials.

All of the asphaltic samples’ spectra exhibit similar stretching vibrations of classic CH_2_, which is demonstrated by the peaks within the range of 2854–2932 cm^−1^.

The peak due to carbonyl C=O group (i.e., carboxylates, ketones, and/or anhydrides) near 1700 cm^−1^ is prominent in the spectrum of NA and appears with no or a very weak intensity in the spectrum of PA; indicating that NA was much more severely oxidized than PA after a prolonged exposure to atmospheric air. This observation is fully in line with the results of elemental analysis (NA oxygen 1.38 wt %; PA oxygen 0.29 wt %).

The band at 1034 cm^−1^ may result from the sulfoxide S=O stretching vibrations. Also, the peaks at 872 cm^−1^, 810 cm^−1^, and 748 cm^−1^ may relate to the out-of-plane bending vibrations of C‒H in phenyl [8]. The sharp peak located at 722 cm^−1^ in the PA spectrum can be associated with long, straight-chain methylene. Overall, the NA contains polar groups such as carbonyl and some clay minerals as observed from the IR spectrum. It is also constituted of minor complex components, which have distinct chemical functional groups.

### 3.5. Raman Spectroscopy (RS)

**Figure 4:**
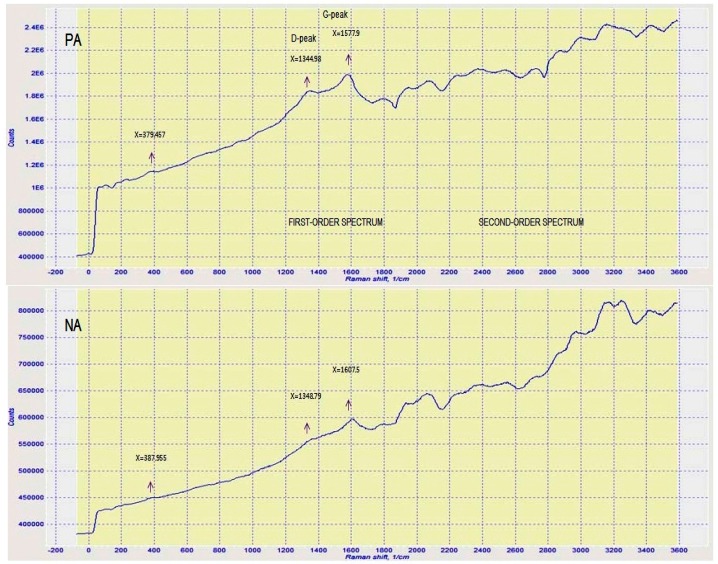
Raman spectra of PA and NA, showing the main Raman features; the D and G bands.

One of the most influential and efficient methods for characterizing asphaltic materials is Raman spectroscopy [9,10]. This method is, therefore, applied here to analyze the characteristics of PA and compare it with NA. The same has been demonstrated in Figure 4, which presents the Raman spectrum within the Raman shift range of 200~3600 cm^−1^. This spectrum demonstrated two first-order characteristic bands of all the asphaltic materials. The first is the D (defect) band at 1344.98~1348.79 cm^−1^ and the second is the G (graphite) band at 1577.90~1607.50 cm^−1^ of graphitic carbon. Whereas the emergence of the D band is due to disordered structures or defects in the carbon, the G band originates from the tangential stretching vibrations of the aromatic C‒C bonds. It is observed from Figure 4 that, in NA, the D and G bands are feebler than those of PA. It is inferred from this revelation that NA has worse order than PA. The analysis results of TLC-FID and elemental analysis data showed that NA has a high concentration of saturates (14.17 wt %) and about 1.38 wt % oxygen, where the higher amounts of oxygen atoms are bound to aliphatic carbon. Therefore, as is evident from these analyses, the higher oxygen content and aliphatic structure have resulted in higher disorder in the structure of NA as compared to PA.

There are various bands that have emerged in the second-order Raman spectra of asphaltic materials at ~2450, ~2695, ~2735, ~2950, and ~3248 cm^−1^. Such bands are allocated to both overtone scattering and combination scattering. Figure 4 shows the same, where all of the asphaltic materials display a weak Raman band within the range of 2500~2800 cm^−1^; this attribute agrees to the overtone of the D band. D band is originally referred to as the G' band as it is symmetric and is apparent in the second-order Raman spectra of crystalline graphite. It is also referred as the 2D or D* band by several researchers [11]. The subtle band of 383 cm^−1^ reveals the amorphous sp^3^-bonded carbon.

### 3.6. Nuclear Magnetic Resonance Spectroscopy (^1^H-NMR)

**Figure 5:**
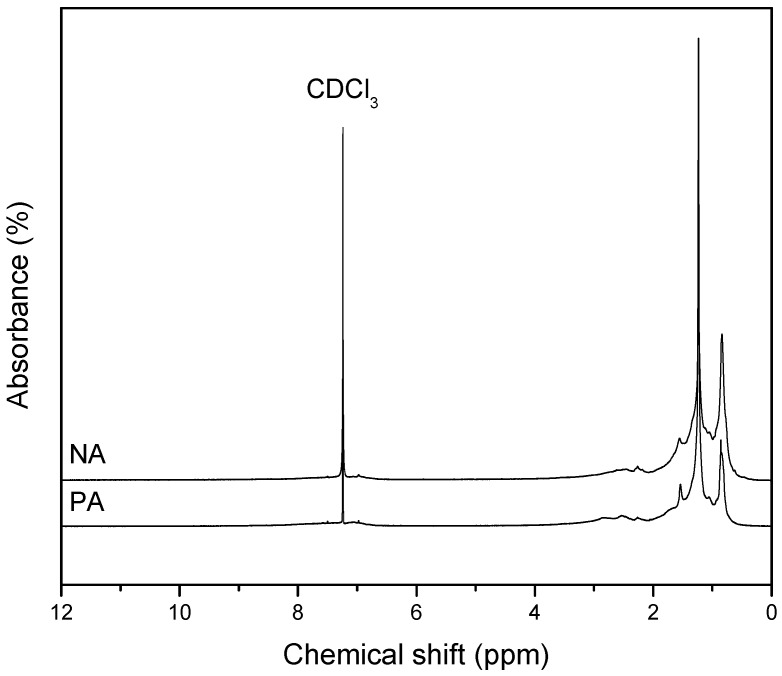
^1^H-NMR spectra of NA and PA.

**Figure 6:**
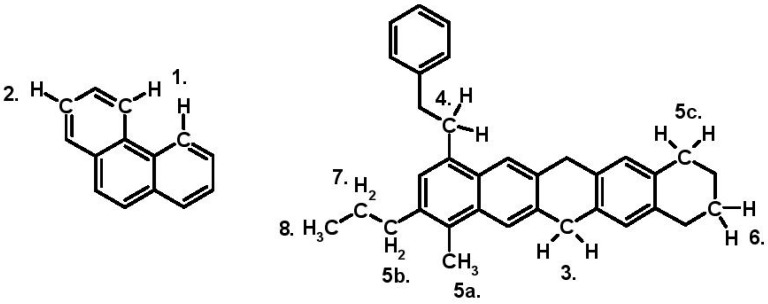
Illustration of types of hydrogen.

Figure 5 gives the proton NMR spectra of varied asphalts. Also, Table 4 and Figure 6 present the positions of the chemical shifts [12]. The distinct types of protons in the asphalt samples are analyzed in terms of their relative proportions through the integration of the spectra. The same are presented in Table 3.

PA and NA are found to exhibit similar spectra, as is evident in the studies of ^1^H-NMR (Figure 5). However, the ^1^H-NMR data in Table 3 shows the highly complex and rich nature of NA as compared to PA. 

The deuterated chloroform (CDCl_3_) is presented by the peak of ^1^H-NMR spectra at 7.3 ppm. To obtain this result, each of the asphaltic samples was dissolved for the NMR measurements. 

When considering the ^1^H-NMR spectra, which is donated by protons, the aromatic peaks are easily determined (H_ar_; δ = 6.5~9.5 ppm) from the aliphatic ones (H_al_; δ = 0.5~4.5 ppm). Also, the spectra were rendered more dependable and consistent. Furthermore, the aliphatic peaks were segregated into three types of protons (α, β, and γ) as per their respective positions in the aromatic core: H_α_, δ = 2.0~4.5 ppm; H_β_, δ = 1.0~2.0 ppm; and H_γ_, δ = 0.5~1.0 ppm. However, the borders defined here are approximate as the impact of heteroatoms and metals on peak shifting is ignored. Irrespective of this, the domains described in Table 4 are often utilized.

Table 3 gives the data about the aliphatic hydrogen concentration, which is found to be higher in NA than PA. This revelation shows that NA is composed of molecules that are highly branched. It is worth mentioning that aromatic conjugated compounds not bearing hydrogen atoms cannot be detected by ^1^H-NMR.

An illustration of the types of hydrogen is displayed in Figure 6 below:

Description and chemical shift ranges in ^1^H-NMR is given above (Table 4).

H_ar2_: 1; H_ar1_: 2; H_F_: 3, H_A_: 4; H_α1_: 5a, b, c; H_β2_: 6; H_β1_: 7; and H_γ_: 8.

Hydrogen in α-methyl group: 5a; Hydrogen in α-methylene in alkyl side chain: 5b; and

Hydrogen in α-methylene in hydroaromatic ring (5c).

### 3.7. Ultraviolet and Visible Spectroscopy (UV-VIS)

**Figure 7:**
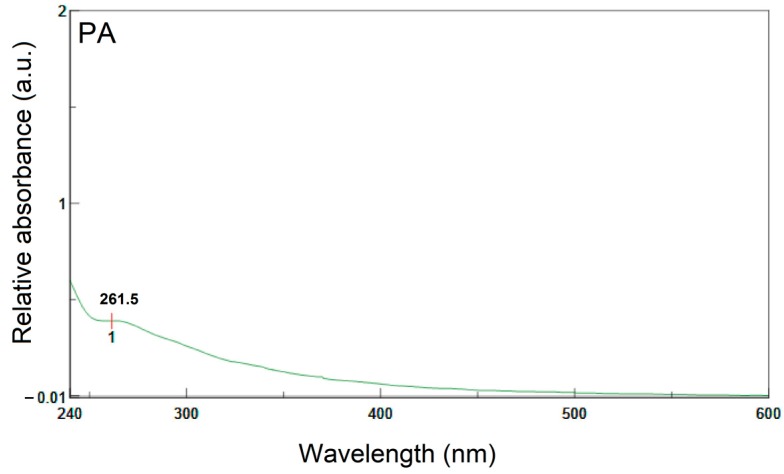

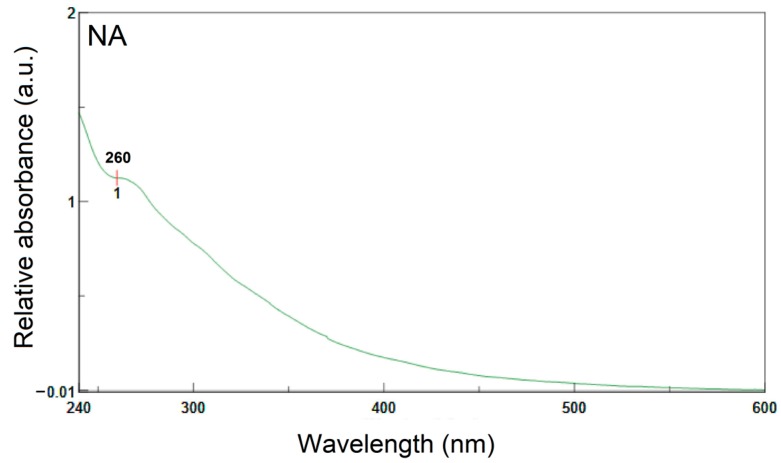
UV-Vis spectra of PA and NA.

Ultraviolet and visible spectroscopy is utilized to examine the structure of the aromatic component in the asphalts. Figure 7 illustrates the UV signal data (absorptivity vs. wavelength) within the range of 240~600 nm by utilizing the UV instrument. The motive behind selecting this UV range is that it has a specific sensitivity to fractions with high contents of aromatic molecules. As per the literature [13], it was observed that the onset wavelength of absorption peak at 270, 320, 380, 470, and 580 nm were of benzene, naphthalene, anthracene, tetracene, and pentacene, respectively. Near 260 nm of the region, an absorption band with an extended wavelength tail is highly significant, as presented in Figure 7. This signifies that the components equipped with benzene rings are dominant for all asphaltic materials. While comparing the spectrum of PA with NA, it was observed that the PA spectrum exhibits weaker absorption band at 261.5 nm. The same is depicted in Figure 7. The findings suggest that NA is equipped with a higher concentration of conjugated systems and/or larger chromophores as compared to PA. To conclude, the UV-Vis spectra have indicated a similar composition of aromatic rings in the aromatic nuclei of both the asphalts. 

### 3.8. X-ray Diffraction (XRD)

**Figure 8:**
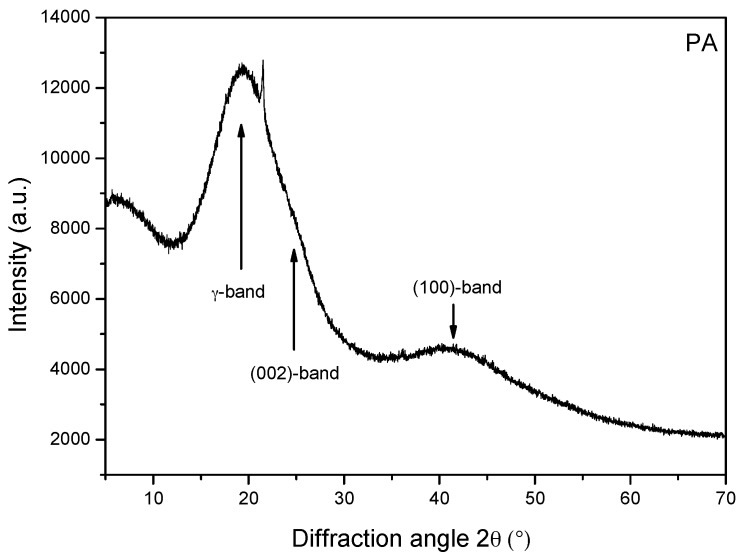
X-ray diffractogram of PA.

**Figure 9:**
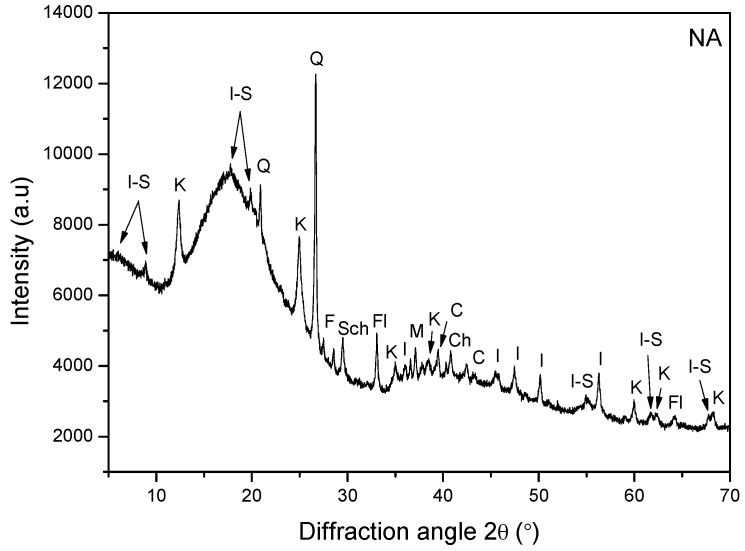
X-ray diffractogram of NA. I-S: illite-smectite; K: kaolinite; Q: quartz; F: feldspar (K-component); Sch: schertelite; M: montmorillonite; I: illite; C: calcite; Fl: fluorapatite.

Figure 8 and Figure 9 demonstrate the X-ray diffraction patterns of PA and NA, respectively. The wide peaks at 18°~26° and 42° in Figure 8 represent the X-ray diffractogram of PA. The γ-peak appears at around 2θ = 18.90° because of the aliphatic chains or condensed saturated rings. The peak settled at approximately 2θ = 23.20° is known as the graphene band or (002)-band. The formation of the graphene band is emerged by staging the aromatic molecules existing in the asphaltenic structure. At the 2θ value (42.39°), a weak band is formed, which is due to the influence of first (100) nearest neighbors in the ring structure [14,15].

The NA material is rendered sticky due to the presence of organic mineral substances. The features of resilience and toughness of asphalts is obtained with the help of organic substances present in it, as it provides the characteristics of binding. H_2_O_2_ digestion was deployed to evaluate the organic fraction of NA. It was observed to be 91.17 wt %. Figure 9 demonstrates the XRD pattern of NA. This figure elucidates the major crystalline components composing the bulk sample of NA. After kaolinite and illite, quartz is found to be the clay material that is present in the greatest abundance. Lower amounts of smectite, calcite, montmorillonite, fluorapatite, and schertelite were also detected. As per this analysis, the mineral composition of the NA is assessed to be over 95% quartz (SiO_2_) and a few percent Feldspars (K-component; KAlSi_3_O_8_). On the other hand, the XRD pattern of PA did not show peaks symbolizing the crystalline structure. 

Compared to the FT-IR technique, X-ray diffraction is a powerful tool in the identification and characterization of minerals in NA. The bulk of the clay fraction of many natural asphalts is crystalline, but clay particles are too small for optical crystallographic methods to be applied. Therefore, XRD has long been a mainstay in the identification of clay-sized minerals in natural asphalts. It is interesting to note that fine mineral matter is expected to strengthen the bitumen in the NA and impart hardiness and enhance pavement surface properties (better tire–road surface interaction), if is added as a modifier in paving-grade asphalt cements. 

### 3.9. Scanning Electron Microscopy (SEM)

**Figure 10:**
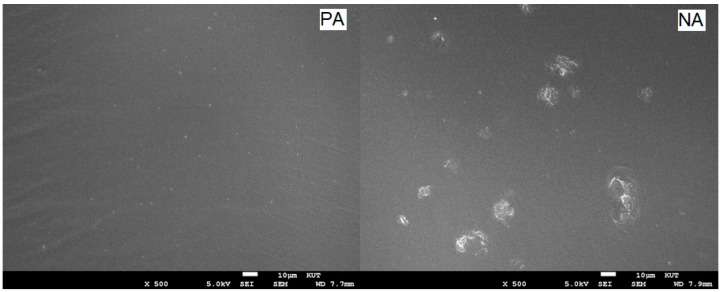
SEM images of PA and NA (showing very fine (clay) particles dispersed into the asphaltic phase), magnification ×500.

A scanning electron microscope (SEM) uses a focused beam of high-energy electrons to generate a variety of signals at the surface of solid specimens. The signals that derive from electron–sample interactions reveal information about the sample including external morphology (texture), chemical composition, crystalline structure, and orientation of materials making up the sample. Figure 10 presents the SEM inspections of asphalt microstructures, indicating some minor differences. The figure shows the broad sizes of unevenly distributed clay particles on a smooth surface as representing NA. The other image, which is homogeneous and uniform, is a representation of the PA sample. As seen in the figure, the PA sample has no clay contamination. This observation is in good agreement with the data of FT-IR and X-ray diffraction.

### 3.10. Thermogravimetric Analysis (TGA)

To acquire and select the best attributes and measurements for asphalt products, it is imperative to gauge the thermal stability of the asphalt materials pertaining to their weight loss due to volatilization. This is an essential property that needs consideration when generating high-performing asphalts for particular applications.

**Figure 11:**
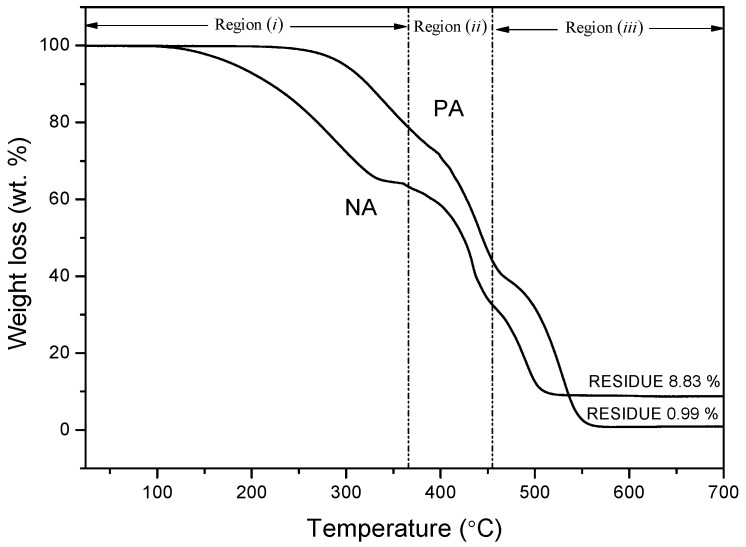
TGA curves of PA and NA.

**Figure 12:**
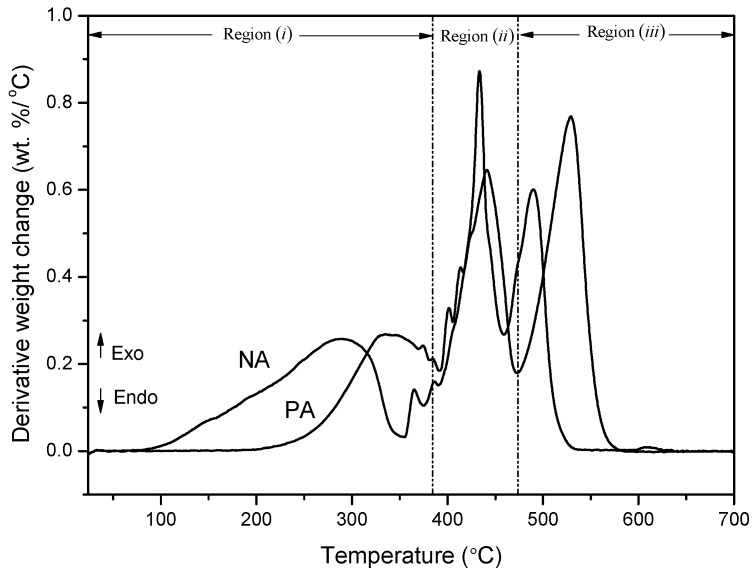
DTA curves of PA and NA.

Thermogravimetric analysis was performed to comprehend the thermal behavior of the asphaltic materials. Figure 11 and Figure 12 represent the TGA (Thermogravimetric analysis) and DTA (Differential thermogravimetric) curves acquired at the heating rate of 10 °C/min, respectively.

Table 5 presents the onset and the offset temperature of thermal degradation (T_onset_, T_offset_), yield of carbonaceous residue, and the maximum decomposition temperature (T_max_) at 700 °C.

When the temperature is subjected to an increase, the tendencies of thermal decomposition of the two asphaltic materials are observed to be similar. Up to at least 180 °C (TGA curve), the samples are found to be stable, after which mass loss occurs. The analytical findings reveal that both PA and NA exhibit different weight loss behavior within a temperature range of 25 °C to 700 °C for 1 h and 13 min. 

After subjecting the samples to high temperatures, it was observed that NA lost large amounts of weight as compared to PA. This indicates that NA has low thermal stability among its molecules. This is in accordance with the elemental analysis and TLC-FID data (Table 1 and Table 2). Also, when considering that H_2_ and CH_4_ evolution is the reason behind higher weight loss during heating [16], the asphalt with the higher hydrogen content (i.e., NA) has a larger weight loss.

As can be seen in Figure 11, the linear superposition of the component reactions in asphalt corresponds to the TGA curves with significant peaks and shoulders. As mentioned before, the asphalt components are segregated into four categories of SARA composition: saturates, aromatics, resins, and asphaltenes [17]. Each of these components has different combustion activity (S + A > R > A) [18], thus each component reaction may correspond to a single mass loss peak.

For PA and NA, at temperatures of 333.3 °C, 441.62 °C, and 546.62 °C; and 286.63 °C, 433.30 °C, and 489.96 °C, respectively, the three major exothermic events are easily differentiated. The existence of these three events is due to the susceptibility of present organic material to igniting in the atmosphere. After the completion of the thermal treatment process, the NA sample had a charred residue of 8.83 wt %, while PA had 0.99 wt % of residue. Such a revelation indicates that NA is constituted of mineral materials that are transformed into ash during combustion.

The three pronounced regions of weight loss, i.e., (*i*), (*ii*), and (*iii*), are evident in Figure 11.

Region (*i*) is formed due to the radical polymerization reaction, whereby the oil content comprising primarily saturates and aromatics is subjected to distillation and combustion [19]. As the oil content has low molecular weight and pyrolysis temperature, they are removed at a low temperature.

In region (*ii*), the peripheral functional groups and heteroatom bonds are fragmented, the resins are oxidized and dehydrogenated, and then asphaltenes and char are generated in the cracking/polymerization reactions. The weight loss of asphalts in this region is due to these reactions. The second peak (405~500 °C) is attributed to the release of secondary volatiles and char combustion. 

The last region (*iii*) is reflected in the second mass loss peak in the TGA peak, within the temperature range of 530~680 °C. In this stage, the aromatic molecules show a steady increment, asphaltenes decompose [20], and char is formed due to the condensation of free radical molecules. The char, which is found to be comparatively stable, is eventually burned.

### 3.11. Differential Scanning Calorimetry (DSC)

**Figure 13:**
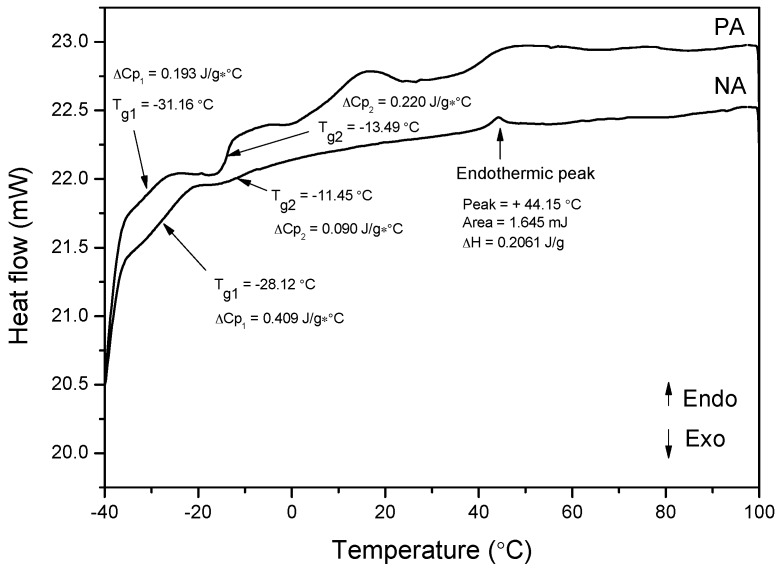
DSC curves of PA and NA.

The asphalts are composed of a combination of distinct hydrocarbons with varied chemical structures and molecular weights. Therefore, it is crucial to comprehend their compositions and their impact on determining the complete thermal behavior of asphalts. Figure 13 demonstrates the DSC scans of both PA and NA.

In the temperature range of −40 °C to 0 °C, asphalts exhibit a broad glass transition. When asphalts are subjected to the DSC scans, different melting peaks or shifts within the peak positions due to variant temperatures are observed, which correspond to the varied shapes of asphalts. There are two distinct glass transition temperatures exposed through the DSC curves, namely, the upper glass transition (T_g2_) and the lower glass transition (T_g1_). Whereas T_g2_ is obtained from the maltene–asphaltene interphase region, T_g1_ is obtained from the maltene phase [21]. When NA is cooled or heated, the crystallites melt and generate a small endothermal peak (T = +44.15 °C) next to the glass transition (T_g2_ = −11.45 °C).

Both PA and NA have T_g (onset)_ near −36.5 °C. The low molecular weights of the materials in the asphaltic fractions are the facilitator of the onset of T_g_. Nonetheless, the differences between their major glass transition temperatures are dissimilar. When comparing PA with NA, PA has a lower, weaker glass transition (T_g1_ = −31.16 °C) than NA, which has a high glass transition (T_g1_ = −28.12 °C). The reason behind this characteristic is the differences in their chemical structure, molecular weight, and distribution. NA has a high concentration of saturates (14.17 wt %) and other sulfur-containing compounds with an average molecular weight of 252 Da, which makes it a black sticky material.

In the case of PA, it has approximately 328 Da of molecular weight along with aromatics in multi-ring structures, 4.40 wt % paraffin, and 18.97 wt % naphthene aromatics. The high molecular weight and inflexible nature of the multi-ring molecular structures in PA result in its greater T_g_ transition.

T_g_ is defined as the temperature at which all the molecular translational motion of the asphalt freezes, which leads to the rigidness of the material at or below this temperature. The T_g_ is related to the low-temperature performance of asphalts. PA is found to have a more brittle nature in low temperature (approximately between −40 and 0 °C) as compared to NA.

The endothermic peaks are more evident in PA than NA. Two major melting peaks are detected in the PA’ DSC thermogram that relate to the dissolution of crystallized fractions in the hydrocarbon matrix. Also, there is a small peak emerging between these two endothermic peaks. 

When heating the asphalts to the wax dissolution, Lesueur (2009) [22] explained the wide endothermic effects occurring between −30 °C and 100 °C in several naturally wax- (or saturate-) encompassing binders. Hence, the highly visible broad endothermic band up to 100 °C in all the asphaltic samples relates to the melting of diverse naturally occurring waxes existing in the asphalt [22,23].

### 3.12. Physical and Rheological Analysis

Table 6 gives the data pertaining to different physical and rheological properties, comprising penetration, softening point, ductility, and viscosity.

#### 3.12.1. Penetration

The major objective of deploying a penetration test is to categorize asphalt cements for the purpose of detecting and acquiring the desired materials. However, it can be used for other purposes as well, such as detecting extended heating of asphalts placed within the storage tanks. It is also useful in evaluating the properties of asphalts, which defines how the respective material has corresponded to time and weathering after it is removed from the pavement [24]. As seen in Table 6, NA has a greater value of penetration, i.e., 147.00 dmm, than PA, which has a value of 63.33 dmm. For avoiding cracks in pavements, soft NA is utilized in the construction of pavements during colder climates and PA is utilized in hot climates.

#### 3.12.2. Softening Point

The softening point of road asphalt is defined as the temperature at which the asphalt transforms from its solid state to a definite liquid state due to the application of some external force and heat. It can be related to the temperature susceptibility of asphalt at high temperature changes. At high temperature, asphalt has less resistance to internal share force. Hence, the asphalt is unable to resist an applied force. As the softening point increases, the deformation of the asphalt decreases due to its increase in resistivity towards high temperatures [25]. Table 6 depicts the softening point of both PA and NA. From the table, it can be seen that PA has a higher softening point (49.00 °C) than NA (40.00 °C). Also, when the softening point is increased, the C/H ratio including the degree of aromatization also increases. The presence of compounds (saturates) with lower molecular weight is a major facilitator in increasing the softening point. Also, the rupture in the double bonds and straight chains, and the polymerization reactions of the radicals formed under the catalytic effect in the asphalts result in a higher softening point.

#### 3.12.3. Ductility Test

The purpose of conducting a ductility test is to evaluate the resistivity of asphalt towards cracking and raveling. It is known that high-ductility asphalts have superior flexibility and tenacity. Similarly, asphalts with lower ductility have a higher tendency to crack under heavy loads or severe temperature variations. The procedures of refining and consistency have great influence on the ductility of the asphalts [24]. As seen in Table 6, after performing the ductility test, it was observed that both PA and NA display high ductility values (>140 cm) (Table 6). Therefore, this revelation suggests that both the asphalt materials will provide good performance during the service life of pavement. 

#### 3.12.4. Viscosity

Viscosity plays a significant role during the manufacturing of asphalt, which comprises distinct stages such as transportation, laying, mixing, and compaction. Depending on the binder, the ranges of viscosity are determined at each of the abovementioned stages [26]. Generally, at a high temperature of 135 °C, the viscosity is applied for evaluating workability, as per the Superpave specification [27]. Also, depending on the grade or viscosity, the asphalts are kept in asphalt plants at temperatures between 149 °C and 177 °C for convenience of pumping [28]. Hence, the current research selected the temperatures of 135 °C and 180 °C for the purposes of viscosity testing. The results revealed in Table 6 suggest that PA has greater viscosity values as compared to those of NA. On this basis, varied workability and storage temperatures are anticipated from the tested asphaltic materials. 

## 4. Conclusions

The current research utilized petroleum asphalt (PA) from an anonymous refining company in South Korea, which was inspected by deploying extensive analytical and rheological techniques to characterize the asphaltic sample. To further estimate the findings and confirm their placement in the framework of the enhanced materials, structural comparisons with natural asphalt (NA) were conducted. The natural asphalt was acquired from Utah, USA. The results obtained from the examination of the asphalts through the battery of analytical techniques were found to be harmonious and comprehensible in terms of the known origins of the samples.

The major findings of the study drawn from the analysis of the data are portrayed below:

- The results of this study revealed a clear distinction between the elemental compositions of PA and NA.
- The SARA composition and the colloidal index stability were highly affected by the chemical composition of the feedstock and the technology of asphalt production.
- Whereas the average value of the molecular weight of PA was found to be 328 Da, it was 252 Da for NA. Therefore, the compound types existent in the asphalts have diverse molecular weight distribution.
- UV-Vis spectroscopy indicates the existence of more highly conjugated systems in NA than in PA.
- NA has a lower carbon aromaticity than PA as observed from the analyses of FT-IR, Raman, and NMR tests. Also, NA has comprehensive alkylated aromatic structures with large amount of oxygenates.
- Whereas clay minerals are present in NA, they are absent in PA. This result was obtained from the FT-IR, XRD, and SEM analyses.
- Thermogravimetric analysis performed in air atmosphere indicated that the PA sample was thermally more stable than the NA sample.
- When performing the differential scanning calorimetry of NA and PA, the former binder demonstrated a narrow endothermic peak at about 40~50 °C, while the latter displayed two broad endothermic bands at 14.50 °C and 48.5 °C. This revelation corresponds to the melting of different crystallizing wax fractions present in the asphalt.
- Due to the different exhibition of chemical composition and structure in both the asphalts, PA and NA performed differently in the rheological tests.

It is strongly recommended that further work should be undertaken to investigate the mechanical properties of asphalt cement binders (and their relationship to pavement performance. Future work may involve the use of different test methods such as forced vibration co-axial shear test (FVAST), dynamic shear rheometer (DSR), bending beam rheometer (BBR), and pressure aging vessel (PAV), etc.

## Figures and Tables

**Table 1:** Elemental analyses of asphaltic materials.

Elements and Ratios	Asphalt	
	PA	NA
Carbon, wt %	87.66	79.01
Hydrogen, wt %	10.14	9.11
Nitrogen, wt %	0.44	0.95
Sulfur, wt %	6.29	0.72
Oxygen, wt %	0.29	1.38
H/C, atomic ratio	1.38	1.38
Organic matter, wt %	99.01	91.17
Inorganic matter, wt %	0.99	8.83

**Table 2:** TLC-FID generic fractions of asphaltic materials.

Asphalt	Saturates wt %	Aromatics wt %	Resins wt %	Asphaltenes wt %	I_C_^†^
PA	4.40	18.97	23.55	53.07	1.35
NA	14.17	13.92	23.36	48.52	1.68

**^†^** I_C_ = (Saturates + Asphaltenes)/(Resins + Aromatics).

**Table 3:** Peak areas as a percentage of total protons for the ^1^H-NMR spectra of PA and NA.

Asphalt	Hydrogen Distribution, %			
	H_α_	H_β_	H_γ_	H_ar_
PA	15.72	66.13	18.15	10.81
NA	13.75	56.97	29.28	8.21

**Table 4:** Proton NMR chemical shifts (ppm) relative to Tetramethylsilane (TMS) [12].

Chemical Shift Range (ppm)	Symbol	Description
9.5–8.36	H_ar2_	Aliphatic hydrogens in sterically hindered positions, highly pericondensed polycyclic aromatic compounds (PACs), next to heteroatoms and some hydrogens joined to nitrogen
8.36–6.3	H_ar1_	All other aromatic hydrocarbons
4.5–3.69	H_F_	Aliphatic hydrogens in methylene groups α to two aromatic rings
3.69–3.0	H_A_	Aliphatic hydrogens in methylene groups α to an aromatic ring and β to another
3.0–2.0	H_α1_	Aliphatic hydrogens in methyl or methylene groups α to an aromatic ring which can also be attached in γ position or further to another or the same aromatic ring
2.0–1.6	H_β2_	Alicyclic hydrogens in β position to an aromatic ring
1.6–1.0	H_β1_	Aliphatic hydrogens in methyl or methylene groups β to an aromatic ring
1.0–0.5	H_γ_	Aliphatic hydrogens in methyl or methylene groups γ to an aromatic ring

**Table 5:** TGA and DTG thermogram data of PA and NA loading at a heating rate of 10 °C/min.

Asphaltic Samples	T_onset_ (°C)	T_offset_ (°C)	T_max_ (°C)	Residue at 700 °C (wt %)
PA	286.63	548.28	333.30/441.62/546.62	0.99
NA	189.96	504.99	286.63/433.30/489.96	8.83

Tonset, the onset temperature of thermal degradation (°C); Toffset, the offset temperature of thermal degradation (°C); Tmax, the maximum decomposition temperature (°C).

**Table 6:** Conventional rheological properties for PA and NA.

Property	Asphalt	
	PA	NA
Penetration at 25 °C, 1/10 mm (dmm)	63.33	147.00
Softening point, (°C)	49.00	40.00
Ductility at 25 °C, (cm)	>140	>140
Viscosity at 135 °C, (Pa·s)	0.50	0.35
Viscosity at 180 °C, (Pa·s)	0.10	0.08
